# Treatment Limitations and Missing Information in Peritoneal Metastatic Gastric Cancer

**DOI:** 10.3390/cancers18091336

**Published:** 2026-04-22

**Authors:** Beate Rau, Franziska Köhler, Annika Kurreck, Safak Gül, Alexander Arnold, Uli Fehrenbach, Resa Puffert, Florian Lordick, Fabian Kockelmann, Thomas Wirth

**Affiliations:** 1 Department of General, Visceral and Transplant Surgery, Hannover Medical School, Carl-Neuberg-Str. 1, 30625 Hannover, Germany; koehler.franziska2@mh-hannover.de (F.K.); puffert.resa@mh-hannover.de (R.P.); kockelmann.fabian@mh-hannover.de (F.K.); wirth.thomas@mh-hannover.de (T.W.); 2 Department of Surgery, Universitätsmedizin Berlin, Corporate Member of Freie Universität Berlin and Humboldt Universität zu Berlin, 10117 Berlin, Germany; safak.guel@charite.de; 3 Department of Haematooncology, Universitätsmedizin Berlin, Corporate Member of Freie Universität Berlin and Humboldt Universität zu Berlin, 10117 Berlin, Germany; annika.kurreck@charite.de; 4 Institute for Pathology, Universitätsmedizin Berlin, Corporate Member of Freie Universität Berlin and Humboldt Universität zu Berlin, 10117 Berlin, Germany; alexander.arnold@charite.de; 5 Institute for Radiology, Universitätsmedizin Berlin, Corporate Member of Freie Universität Berlin and Humboldt Universität zu Berlin, 10117 Berlin, Germany; uli.fehrenbach@charite.de; 6 Department of Medicine (Oncology, Gastroenterology, Hepatolgy, and Pulmonology), Comprehensive Cancer Center Central Germany (CCCG), University of Leipzig Medical Center, 04103 Leipzig, Germany; florian.lordick@medizin.uni-leipzig.de

**Keywords:** gastric cancer, peritoneal disease, HIPEC, CRS, peritoneal metastatic gastric cancer

## Abstract

**Background/Objectives**: Peritoneal metastasis represents the most frequent and prognostically unfavorable metastatic pattern in gastric cancer, largely due to limited sensitivity of conventional imaging, delayed diagnosis, and insufficient response assessment. The aim of this review is to provide an overview of the current evidence on the diagnosis and treatment of gastric cancer with peritoneal metastases and to address current treatment limitations and options. **Methods**: This review was designed as a narrative review and is based on an extensive literature search in established databases. **Results**: Systemic chemotherapy remains the cornerstone of palliative treatment, improving the survival and quality of life compared with the best supportive care; however, outcomes in peritoneally metastatic disease remain poor. Advances in molecularly targeted and immune-based therapies have extended survival in selected patient populations, yet favorable molecular profiles are mainly unknown in peritoneal metastases. Staging laparoscopy and semi-quantitative assessment using the Peritoneal Cancer Index (PCI) are therefore essential for accurate diagnosis, prognostication, and treatment selection. Growing evidence from retrospective studies, multi-institutional cohorts, and selected randomized trials suggests that a multimodal approach—combining systemic therapy with intraperitoneal or bidirectional chemotherapy—may improve survival and quality of life. In carefully selected patients whose primary gastric tumor and peritoneal lesions respond to systemic treatment, complete cytoreductive surgery (CRS) followed by hyperthermic intraperitoneal chemotherapy (HIPEC) may further enhance outcomes and, in rare cases, achieve long-term survival. These potential benefits appear to be limited to highly selected patients with a low peritoneal tumor burden (PCI ≤ 6–7), positive cytology, good performance status, controlled extraperitoneal disease, and a high likelihood of achieving complete macroscopic cytoreduction (CC-0). **Conclusions**: Although the treatment intent in metastatic gastric cancer remains primarily palliative, carefully selected patients with limited peritoneal metastases may benefit from intensified multimodal treatment strategies when managed in specialized centers. Interdisciplinary evaluation, accurate staging, and individualized treatment planning are essential to optimize outcomes in this challenging disease setting.

## Simple Summary

This narrative review summarizes current evidence on the diagnosis and treatment of gastric cancer with peritoneal metastases and provides recommendations for its management based on the available evidence. Although systemic therapy remains largely palliative, carefully selected patients with low peritoneal tumor burden and a high likelihood of complete cytoreduction (CC-0) may achieve meaningful survival benefits from aggressive multimodal approaches including intraperitoneal localized chemotherapy in specialized centers.

## 1. Introduction

The treatment of gastric cancer is determined by several established oncological parameters, namely the tumor infiltration depth (T), local lymph node involvement (N), and the presence of distant metastases (M). In early gastric cancer, curative treatment consists of oncologic surgical resection of the primary tumor including regional lymph node stations, typically performed as a D2 lymphadenectomy. This approach leads to long-term cure in most patients.

In locally advanced gastric cancer, treatment decisions are not yet based on additional molecular and pathological tumor characteristics. However, in metastatic disease they are essential for selecting the most appropriate treatment strategy. Biomarkers such as HER2, PD-L1, microsatellite instability, and Claudin 18.2 have become part of modern treatment algorithms [1].

The treatment of synchronous metastatic gastric cancer remains complex. Therapeutic decisions are based mainly on several critical factors:

(1) The anatomical location of metastases (e.g., lung, liver, non-regional lymph nodes, bone, or peritoneum);
(2) The extent of metastatic disease, including the number of involved organs and the quantitative tumor burden especially of peritoneal disease;
(3) The molecular and biological tumor profile.

Among all metastatic patterns, peritoneal metastases are consistently associated with the poorest prognosis. This unfavorable outcome may partly be explained by the biological aggressiveness but also by substantial diagnostic limitations. Small peritoneal nodules frequently escape detection by standard imaging modalities, leading to delayed diagnosis and limited therapeutic options at the time of detection. (Table 1 and Table 2). The currently available data from DRAGON-01, PRECISE-SG1, and PERISCOPE II should be interpreted with caution because they are based on abstract reporting, interim analyses, or incomplete publication formats. These findings are clinically relevant and hypothesis-generating, but they do not justify strong treatment recommendations at present.

## 2. Material and Methods

This review was conducted as a structured narrative review. A comprehensive literature search was performed in PubMed/MEDLINE, Embase, and the Cochrane Library for articles published over the past decade (2015–2025). The search strategy combined terms related to metastatic gastric cancer and peritoneal disease, including “gastric cancer”, “gastric adenocarcinoma”, “peritoneal or distant metastasis”, “peritoneal carcinomatosis”, “positive peritoneal cytology”, “cytoreductive surgery”, “HIPEC”, “PIPAC”, “NIPS”, “intraperitoneal chemotherapy”, and “bidirectional chemotherapy”. A total of 35 articles were retrieved and formed the basis of this review.

Articles were considered eligible if they addressed the diagnosis of metastatic gastric cancer, staging, systemic therapy, surgical management, or intraperitoneal treatment of gastric cancer with peritoneal metastases or positive cytology. Priority was given to randomized controlled trials, prospective studies, multicenter cohort studies, meta-analyses, and international guidelines. Relevant retrospective series were included when higher-level evidence was limited. Case reports, very small case series, duplicate publications, and studies without clear relevance to peritoneal metastatic gastric cancer were excluded.

The evidence was synthesized descriptively and evaluated based on the study design, sample size, clinical relevance, risk of selection bias, and completeness of reporting. Data reported only in abstract form or from interim analyses were considered exploratory and interpreted with caution.

## 3. Pathogenesis, Prognosis and Therapeutic Challenges of Peritoneal Metastases of Gastric Cancer

### 3.1. Clinical Impact and Prognosis of Peritoneal Metastatic Gastric Cancer

Gastric cancer remains one of the leading causes of cancer-related mortality worldwide. In 2020, the global incidence was estimated at approximately 1.09 million new cases, with nearly 770,000 deaths [11]. Peritoneal metastases occur frequently in locally advanced gastric cancer and are observed in approximately 37% of patients during the disease course.

The prognosis of patients with histologically confirmed peritoneal metastases is extremely poor. Synchronous peritoneal metastases are associated with a significantly worse outcome compared to with isolated metastases at other sites, such as the liver, lung, or distant lymph nodes, in the absence of peritoneal involvement [12]. Isolated peritoneal metastases are identified synchronously in approximately 30% of cases and metachronously in up to 50% of patients.

Systematic chemotherapy remains the standard of care in metastatic gastric cancer and is associated with a median overall survival of approximately 12–18 months, depending on patient characteristics and tumor biology [13]. However, due to major diagnostic challenges in identifying isolated peritoneal metastases and in assessing the response to treatment, very few randomized trials have specifically evaluated systemic therapy in this subgroup shown in Table 1. As a result, patients with isolated peritoneal metastases are typically grouped together with other metastatic patterns and treated accordingly.

### 3.2. Biological Development of Peritoneal Metastases

The development of peritoneal metastases in gastric cancer follows a series of well-defined biological steps. Initially, gastric cancer cells invade the layers of the gastric wall and penetrate the serosa, thereby gaining access to the peritoneal cavity. Within the abdominal cavity, tumor cells disseminate and adapt to a microenvironment characterized by hypoxia, acidosis, and limited nutrient availability [5]. Subsequently, tumor cells adhere to the mesothelial layer of the peritoneum and invade through the mesothelial layer and basement membrane into the submesothelial space. In this compartment, cancer cells encounter blood vessels, enabling angiogenesis and the establishment of new metastatic lesions [14].

Tumor cell detachment from the primary tumor occurs via different mechanisms. On the one hand, mechanical exfoliation may result from serosal invasion or tumor perforation. On the other hand, detachment may occur through epithelial–mesenchymal transition (EMT), a dynamic process characterized by loss of epithelial polarity and acquisition of mesenchymal characteristics. Gastric cancer cells undergoing EMT appear to have a higher propensity for peritoneal dissemination and are more frequently associated with diffuse-type histology [15].

### 3.3. Mechanisms of Chemoresistance and Risk Factors for Peritoneal Metastases

Multiple mechanisms contribute to the chemoresistance observed in peritoneal metastases of gastric cancer. A central role is played by altered gene expression patterns in tumor cells. These expression patterns lead to the increased expression of membrane transport proteins that regulate drug efflux, as well as the enhanced intracellular degradation of chemotherapeutic agents [16].

EMT itself represents another important mechanism of chemoresistance. This phenotypic transformation is associated with changes in membrane protein composition and intracellular signaling pathways, resulting in reduced sensitivity to cytotoxic agents [17]. In addition, inhibition of apoptosis represents a major survival mechanism for malignant cells and contributes substantially to treatment resistance [15].

Furthermore, the tumor microenvironment plays an important role in chemoresistance. Peritoneal metastases are characterized by alterations in local conditions, including pH changes, nutrient deprivation, and trace element imbalance. Among these factors, hypoxia appears to be particularly relevant. Hypoxic conditions lead to the altered expression of drug resistance genes and impair the development and distribution of blood vessels, resulting in decreased delivery of systemic antitumoral agents to tumor tissue [18].

In recent years, increasing attention has been directed toward the role of non-coding RNA and exosome-mediated intercellular communication in chemoresistance. Several pathways have been identified in which microRNA and exosomes modulate gene transcription of proteins involved in drug resistance. Notably, Ohzawa et al. demonstrated that specific miRNA profiles in peritoneal exosomes can predict the response to intraperitoneal chemotherapy in gastric cancer [19].

Several clinicopathological factors have been associated with the development of peritoneal metastases in gastric cancer. Based on analyses of large Dutch and German cohorts, peritoneal dissemination appears to be more frequent in male and younger patients, in tumors with non-cardia localization, and in diffuse-type gastric cancer [20]. These findings support the concept that peritoneal metastasis represents a biologically distinct disease entity in metastatic gastric cancer.

## 4. Diagnosis and Staging of Peritoneal Metastases in Gastric Cancer

### 4.1. Conventional Diagnostic Pathways

The diagnostic work-up of gastric cancer includes esophagogastroduodenoscopy (EGD) with documentation of tumor localization and histological confirmation. Endoscopic ultrasound (EUS) complements endoscopy by allowing the assessment of the tumor infiltration depth, regional lymph node involvement, and the presence of ascites.

Cross-sectional imaging using contrast-enhanced computed tomography (CT) or magnetic resonance imaging (MRI) of the chest and abdomen is mandatory for staging and restaging in gastric cancer. CT provides an excellent cross-sectional overview of the local tumor extent, lymphatic spread, and distant organ metastases, and it serves as the primary modality for treatment response assessment before and after systemic therapy. However, accurate TNM T-staging is limited, as early tumor stages (T1 vs. T2) cannot be reliably distinguished due to the insufficient visualization of the individual gastric wall layers on CT. Magnetic resonance imaging (MRI) may provide additional information in selected cases, particularly for assessing the local tumor extent including peritumoral fat infiltration, identifying localized peritoneal carcinomatosis, and for the detection and characterization of liver metastases.

However, despite continuous technical improvements, cross-sectional imaging remains limited for diagnosing peritoneal metastases. Small-volume diseases such as subcentimeter peritoneal nodules and subtle flat infiltrative involvement may be missed, leading to an underestimation of the true peritoneal tumor burden on CT and conventional MRI. Consequently, peritoneal metastases can be radiologically occult and may not be reliably described or quantified based on imaging alone [21].

### 4.2. Limitations in CT-Based Assessment of Peritoneal Metastases

In the evidence report for the German S3 guideline on gastric cancer, the diagnostic value of CT imaging is primarily discussed in the context of liver and lymph node metastases. In the section addressing M staging, peritoneal metastases are mainly described indirectly via the detection of ascites, reflecting the limitations of CT in this setting [22]. Several studies have systematically evaluated the diagnostic performance of CT for detecting peritoneal metastases. Yajima et al. demonstrated that ascites were present on CT in only 15% of patients with peritoneal metastases, with a sensitivity of 51% and a specificity of 97% [23]. Chang et al. reported a strong correlation between ascites volume and detection rate: while peritoneal carcinomatosis was detected in only 25% of patients with ascites volume ≤ 50 mL, detection increased to 100% in patients with up to 300 mL ascites. Notably, 10% of patients with histologically confirmed peritoneal carcinomatosis had no detectable ascites at all [24].

Without considering ascites, the reported CT sensitivity for peritoneal metastases ranges from 6% to 76%, with a median sensitivity of approximately 40%, while the specificity remains high at 92–100%. These wide ranges reflect not only technical limitations but also substantial interobserver variability. In this context, the expertise of the reporting radiologist is highly relevant. Barry et al. demonstrated that the sensitivity for detecting distant metastases increased from 5% when interpreted by non-specialists to 25% when assessed by experienced radiologists, whereas the specificity remained high in both groups [25].

Most imaging studies focus exclusively on the presence or absence of peritoneal disease, while volumetric assessment and response evaluation are rarely addressed.

### 4.3. Limitations of Imaging-Based Therapy Response Assessment

A key consequence of the limited measurability of peritoneal metastases on cross-sectional imaging is that standardized size-based response criteria such as RECIST 1.1 are frequently not applicable. RECIST requires measurable target lesions (≥10 mm on CT), whereas peritoneal carcinomatosis and small peritoneal implants are commonly categorized as non-measurable disease; ascites is explicitly considered a non-measurable finding. Consequently, patients with exclusively peritoneal metastases are often underrepresented in clinical trials using RECIST endpoints, and robust objective imaging-based data on treatment response under systemic chemotherapy in this subgroup remain limited.

In the clinical routine and in many trial settings, the response assessment in peritoneal disease therefore relies on indirect or non-target lesion evaluation, for example, changes in ascites volume or the evaluation of bulky omental caking or confluent peritoneal masses. However, ascites is a late and nonspecific manifestation and do not reliably quantify the peritoneal tumor burden or treatment response; even CT-detected ascites shows limited specificity for peritoneal metastasis and require cautious interpretation in gastric cancer.

### 4.4. The Role of PET Imaging and Further Developments

In selected clinical scenarios, particularly when conventional imaging findings are equivocal or when recurrence is suspected, FDG PET/CT can provide complementary information by depicting metabolically active disease and may reveal otherwise occult extra-peritoneal metastases. However, the diagnostic performance of FDG-PET in gastric cancer is heterogeneous and depends on the tumor biology; diffuse-type, mucinous, and signet-ring cell carcinomas, as well as small-volume peritoneal disease, may show low FDG avidity and can therefore be missed [26,27].

In recent years, PET imaging targeting fibroblast activation protein (FAP) has emerged as a promising diagnostic in gastric cancer. FAPI-PET is based on the radionuclide imaging of FAP, which is highly expressed by cancer-associated fibroblasts within the tumor microenvironment and peritumoral stroma [28].

Several studies have demonstrated that FAPI-PET/CT provides superior tumor delineation, higher tumor-to-background contrast, and improved detection rates compared with FDG-PET. In gastric cancer, FAPI-PET has shown advantages in detecting peritoneal metastases and diffuse-type tumors [29].

A meta-analysis including 523 patients reported a significantly higher sensitivity and specificity of 68Ga-FAPI-PET for detecting primary tumors as well as lymphatic, distant, and peritoneal metastases compared with 18F-FDG-PET [28]. Based on these findings, the authors suggested that FAPI-PET has the potential to replace FDG-PET in the diagnostic algorithm of gastric cancer. Nevertheless, despite its promising diagnostic performance, FAPI-PET has not yet been approved by either the European Medicines Agency (EMA) or the U.S. Food and Drug Administration (FDA), and its use remains limited to research and specialized centers.

### 4.5. Staging Laparoscopy and Its Diagnostic Value

Staging laparoscopy is the most sensitive diagnostic method for identifying occult peritoneal metastases in patients with locally advanced gastric cancer. Despite negative cross-sectional imaging results, peritoneal metastases are identified laparoscopically in 20–30% of patients, particularly those with T3/T4 tumors or diffuse-type histology [30].

For this reason, cross-sectional imaging alone is not adequate for the staging of advanced gastric cancer, and staging laparoscopy has become an important part of the diagnostic standard in many guidelines and centers. In addition, laparoscopy allows histological confirmation of suspicious lesions, a semi-quantitative assessment of tumor burden using the Peritoneal Cancer Index (PCI) and cytological analysis of the peritoneal fluid.

Despite its established value, staging laparoscopy is inconsistently applied in routine clinical practice. As a result, many centers rely exclusively on radiological imaging and ascites detection, leading to underestimation of the peritoneal tumor burden following inadequate patient stratification.

Additionally, patients with positive cytology represent a biologically distinct high-risk subgroup, although cytological conversion has been observed in selected patients undergoing systemic or bidirectional chemotherapy. This raises important questions about how treatment should be stratified and whether intensified locoregional approaches could play a role in carefully selected cases. Therefore, diagnostic laparoscopy is, according to the guidelines, an unmet need in staging of gastric cancer [31].

Restaging laparoscopy after induction chemotherapy is becoming increasingly relevant within conversion-oriented treatment concepts. In patients with initially limited peritoneal metastases, repeat laparoscopy enables an objective reassessment of the PCI, documentation of tumor regression, evaluation of cytological conversion and determination of the feasibility of complete macroscopic cytoreduction. Reliance solely on imaging for response assessment remains problematic due to the absence of measurable target lesions according to RECIST criteria in most cases of peritoneal disease.

Standardized documentation of the peritoneal disease distribution and tumor burden, including systematic assessment of defined abdominal regions, is essential to improve the staging accuracy, the reproducibility across institutions, and appropriate patient selection for cytoreductive strategies.

### 4.6. Diagnostic Implications for Tumor Quantification

The precise quantification of the peritoneal tumor burden is essential for treatment planning, prognostic assessment, and selection of patients for multimodal treatment strategies. Given the limitations of radiological imaging, laparoscopic assessment remains the most reliable method for evaluating peritoneal metastasis, for determining the possibility of cytoreductive surgery, and for indicating further treatment options as well as assessing the response.

This need for standardized intraoperative assessment forms the basis for the development and widespread adoption of the Peritoneal Cancer Index (PCI), which will be discussed in the following subsection.

### 4.7. Classification Systems for Peritoneal Metastasis

Accurate assessment of peritoneal carcinomatosis requires the reliable quantification of the tumor burden for treatment planning and prognostic evaluation. The Peritoneal Cancer Index (PCI), introduced by Sugarbaker et al. in 1996, provides a structured and reproducible method by dividing the abdomen into regions and scoring the lesion size, yielding a cumulative score from 0 to 39 [32].

Earlier systems, such as the Japanese P-classification and its revised P1abc version, as well as the Lyon classification, offer simpler or more anatomically detailed approaches but lack precise information on tumor size and overall burden, limiting their clinical utility. Comparative studies have shown substantial variability in their prognostic value, particularly for cytoreductive surgery [33,34].

Among available systems, the PCI provides the most detailed and clinically relevant assessment. In gastric cancer, a low PCI (≤6) is consistently associated with improved survival and higher benefit from CRS and HIPEC. Owing to its strong correlation with outcomes, anatomical precision, and practicality, the PCI is now the most widely used scoring system for peritoneal carcinomatosis in Western clinical practice [35,36,37,38].

## 5. Clinically Relevant Subgroups in Peritoneal Metastatic Gastric Cancer

**Figure 1:**
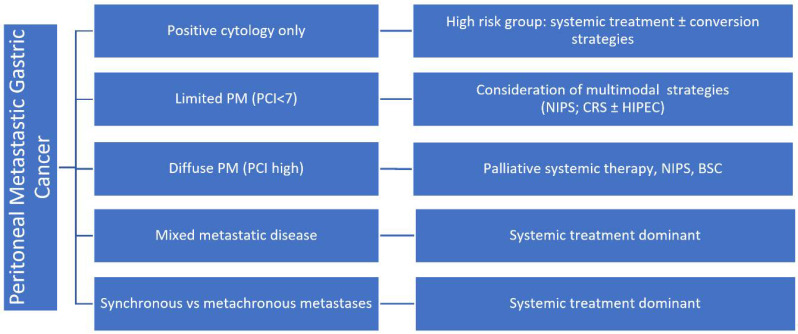
Treatment algorithm for gastric cancer with peritoneal involvement, according to the NCCN Guidelines for gastric cancer (modified), 2025 [39], PM: peritoneal metastases, PCI: peritoneal cancer index; NIPS: normothermic intraperitoneal chemotherapy; BSC: best support of care.

Peritoneal metastatic gastric cancer should not be considered a uniform clinical entity. Preclinical models integrating proteomic, immune, and radiomic analyses of primary tumors have provided important insights into the mechanisms underlying peritoneal dissemination [39]. Furthermore, studies in diffuse-type gastric cancer have shown that the loss of cell adhesion promotes peritoneal spread through interactions with the tumor microenvironment [40]. Therefore, the effectiveness of systemic treatment may vary across clinically relevant subgroups. Patients with positive peritoneal cytology without visible metastases, those with limited peritoneal disease and a low peritoneal cancer index (PCI), diffuse carcinomatosis with high PCI, synchronous versus metachronous presentation, and isolated peritoneal versus mixed metastatic spread may require distinct therapeutic considerations and subgroup-specific recommendations [41]. These categories differ in their biological behavior, resectability, response assessment, and their potential suitability for multimodal treatment (see Figure 1).

## 6. Histological Response Evaluation

Histological assessment of tumor regression following neoadjuvant therapy is an important prognostic parameter in locally advanced gastric cancer. To assess the effectiveness of preoperative therapy, several tumor regression grading systems have been published.

The Becker regression grading system has been established for gastric cancer [40]. It describes tumor regression based on the proportion of remaining viable residual tumor tissue. Grade 3 indicates a prognostically unfavorable situation, as the tumor has shown a poor response to chemotherapy. Different studies demonstrated a strong association between the Becker TRG scores and overall/disease-free survival after neoadjuvant chemotherapy [40,41]. This classification has shown good reproducibility and prognostic discrimination in gastric cancer patients. Its use in large prospective perioperative trials like the FLOT trials demonstrate its role as the preferred system for TRG in gastric cancer in Europe [42]. Despite its wide use in Europe, limitations for the Becker score could include the subjective estimation of the residual tumor percentage, which refers to interobserver variability.

In contrast to the Becker classification, the AJCC/CAP tumor regression grading system was developed as a general applicable grading system and is therefore widely used for different tumor entities in the Anglo-American setting [43]. This grading system describes tumor regression within four different grades, ranging from complete response to no visible treatment effects. The advantage of this score lies in its simplicity and its wide use in different tumor entities. On the other hand, this score is not specific for gastric cancer, with no strength cut-off values, which causes limitations and its inferiority in contrast to more specific systems like the Becker classification [44].

The Japanese Gastric Cancer Association (JGCA) published a histological response grading system, which categorizes tumor regression after neoadjuvant therapy based on the proportion of residual viable tumor relative to the original tumor area, ranging from Grade 0 (no response) to Grade 3 (complete pathological response) [45]. Although the Japanese system subdivides partial responses in more detail, the estimation of the initial tumor extent can be challenging because of fibrosis and other structural alterations after neoadjuvant therapy. The assessment of residual viable tumor, as required by the Becker classification, is often easier and may therefore be more reproducible.

For these reasons, the Becker classification may be preferable for histopathological response in gastric cancer, especially in Western neoadjuvant treatment settings.

## 7. Palliative Treatment Options for Metastatic Gastric Cancer

Systemic therapy is the standard palliative treatment for patients with metastatic gastric cancer. In most clinical trials, this group is analyzed together with patients who have locally advanced unresectable disease, which is generally associated with a poor prognosis. Compared with best supportive care alone, palliative chemotherapy has been shown to improve overall survival and to maintain the quality of life, symptom control, and performance status [46,47]. This benefit was confirmed in a Cochrane meta-analysis including 60 randomized trials and 11,700 patients, demonstrating a mean survival advantage of 6.7 months for patients receiving chemotherapy [48]. Nowadays in selected patients with adjusted treatment according to tumor biology, median overall survival durations of 12–18 months can be achieved [13].

More recently, targeted therapies and immune checkpoint inhibitors have demonstrated improved outcome in selected patient populations. Their use depends on specific molecular tumor characteristics, such as MMR (mismatch repair deficiency), HER2 positivity, PD-L1 status, or Claudin 18.2 expression. Phase III trials such as CheckMate 649, SPOTLIGHT and KEYNOTE-811 reported a median overall survival of 14.4 and 18.2 months in molecularly selected patients [4,5,6]. Information on peritoneal metastases was limited. Only CheckMate 649 reported peritoneal involvement, present in 38% of patients, without providing outcome data for this subgroup (Table 1).

Earlier chemotherapy studies showed similar limitations. In the V325 trial, the addition of docetaxel to cisplatin and fluorouracil (DCF) improved the median overall survival to 9.2 months and achieved a response rate of 37%, at the cost of increased toxicity [49]. Subsequent studies demonstrated that replacing cisplatin with oxaliplatin reduced severe adverse events combined with comparable efficacy, leading to the use of FLOT-like regimens in the palliative setting [50,51]. However, only 33% of patients in this cohort had peritoneal metastases, limiting their relevance for this subgroup (Table 1).

Similar results have been observed in targeted therapy trials. In the ToGA study, the addition of trastuzumab to standard chemotherapy improved the median overall survival from 12 to 16 months in patients with HER2-positive tumors [2]. Peritoneal metastases were not reported as a distinct metastatic site. In contrast, the GASTRIPEC study, which exclusively included patients with histologically confirmed peritoneal metastases, identified HER2 positivity in only 10% of patients [8]. In addition, signet-ring cell carcinomas, which are present in approximately 70% of gastric cancers with peritoneal dissemination, were not specifically addressed in the ToGA trial (Table 1).

The results of systemic therapy trials in advanced gastric cancer cannot be directly extrapolated to patients with isolated or predominant peritoneal metastases. First, peritoneal lesions are often non-measurable according to RECIST criteria, limiting objective response assessment. Second, peritoneal tumor deposits are characterized by impaired vascularization, stromal barriers, hypoxia, and reduced drug penetration, all of which may diminish the efficacy of systemic therapies. Third, many first-line trials have either not reported outcomes for patients with peritoneal disease separately or have included them only within heterogeneous metastatic populations. Consequently, the survival outcomes derived from unselected metastatic cohorts may overestimate the true benefit in patients with peritoneal metastatic disease. In addition, peritoneal metastases frequently arise from distal gastric cancers and are commonly associated with signet ring cell histology or poorly cohesive carcinoma. These tumor subtypes are often characterized by limited actionable molecular alterations, which restricts the applicability of targeted or biomarker-driven systemic therapies.

## 8. Local Treatment Options in Peritoneal Metastatic Gastric Cancer

### 8.1. Palliative Surgery Alone

Palliative surgery may be indicated in selected situations such as bleeding, perforation, or obstruction requiring immediate surgical intervention. In other cases, a potentially curative approach with resection of the primary tumor and limited metastatic disease may be considered.

The AIO-FLOT3 trial reported prolonged survival in patients with limited metastatic disease treated with chemotherapy followed by surgery compared with patients with extensive metastases who did not undergo resection. Only four patients with peritoneal metastases were included. Peritoneal metastases were not adequately characterized, nor was the disease extent quantified using standardized measures such as the Peritoneal Cancer Index (PCI). Moreover, neither a formal cytoreductive surgery concept nor HIPEC was incorporated. Therefore, these results do not support surgery for peritoneal metastatic disease [52].

Similarly, the RENAISSANCE (AIO-FLOT5) trial [3] addressed the role of surgical resection following chemotherapy in patients with limited metastatic gastric or gastroesophageal junction adenocarcinoma. While methodologically more robust as a phase II trial, the study focused on systemic metastases as a collective entity and did not stratify outcomes according to the metastatic pattern or peritoneal tumor burden. Peritoneal metastasis was again treated as a biologically uniform condition, and neither CRS principles nor intraperitoneal chemotherapy were part of the treatment strategy [3]. The results of the trial were presented at ASCO 2024 [53] and not yet published as a full paper. The study showed no benefit of the primary tumor and metastases in metastatic gastric cancer—systemic chemotherapy alone yields the same overall survival outcomes with fewer adverse events and less treatment-related morbidity and mortality. Even more, the authors pointed out that, in a subgroup analysis involving 41 individuals, patients with peritoneal disease showed significantly worse results with a median overall survival of 11.9 months compared to 18.6 months with chemotherapy only. However, only 37% of the randomized patients were treated as supposed; the patients were treated mainly in non-certified centers and without state-of-the-art evaluation of PCI.

These findings do not support a routine intensified surgical approach in metastatic gastric cancer and underscore the importance of careful patient selection, biological stratification, and treatment in experienced centers. At the same time, they do not fully resolve the question of whether highly selected patients with limited peritoneal disease may benefit from CRS-based multimodal concepts (Table 2).

A potential benefit of combined surgical and systemic treatment has also been suggested in a meta-analysis, provided that an R0 resection of the primary tumor and metastases can be achieved [54].

In patients with peritoneal metastases, surgical evaluation should be restricted to specialized centers with experience in cytoreductive surgery (CRS) [55]. Surgical treatment in this setting is reserved for a small highly selected group of patients with limited tumor burden, typically defined by a low Peritoneal Cancer Index (PCI), when complete macroscopic resection appears feasible.

### 8.2. Potentially Curative Cytoreductive Surgery and HIPEC

Hyperthermic intraperitoneal chemotherapy (HIPEC) can be used to eliminate micro metastases during cytoreductive surgery (CRS). In HIPEC, chemotherapeutic agents are heated to 41–43 °C to enhance their cytotoxic effect and improve tissue penetration. The primary benefit of intraperitoneal chemotherapy is the ability to increase the intra-abdominal drug concentrations without inducing systemic adverse effects. The efficacy of cisplatin, mitomycin C, or doxorubicin is increased by hyperthermia; therefore, these agents are most used for this procedure [56]. Retrospective studies have demonstrated an improvement in the median overall survival up to 27.7 months following the combination of CRS and HIPEC [57].

In individual cases, selected patients with good performance status may benefit from a multimodal treatment approach with specific criteria: synchronous peritoneal metastases, isolated peritoneal disease identified by pretherapeutic laparoscopy with PCI < 7, perioperative chemotherapy, response to treatment and the possibility of complete macroscopic cytoreduction.

Nevertheless, given the overall poor prognosis and the complex biological characteristics of gastric cancer subtypes, the treatment of limited peritoneally metastatic gastric cancer remains controversial.

Several cohort studies and registry analyses, together with a limited number of randomized trials, have examined whether an aggressive multimodal strategy combining cytoreductive surgery (CRS) and HIPEC can improve outcomes in patients with peritoneal metastases from gastric cancer. While these data demonstrate that long-term survival is possible in selected individuals, they also reveal substantial limitations, particularly regarding patient selection, treatment-related morbidity, and the lack of survival benefit in controlled settings.

The BIG RENAPE study by Chia et al. included 81 patients undergoing complete cytoreduction followed by HIPEC. A 5-year survival rate of 18% was reported, with 11% of patients achieving a disease-free interval beyond 5 years. However, all long-term survivors had a PCI ≤ 6 and achieved CC-0 resection [38]. This highlights the importance of optimal patient selection. Multivariate analysis further identified synchronous resection as a significant prognostic factor for improved overall survival.

The large single center experience from Osaka [57] reported outcomes of 419 patients treated with bidirectional neoadjuvant chemotherapy, CRS and HIPEC. A 10-year survival rate of 8.3% after CC-0 resection is promising but must be interpreted in the context of strict selection. Again, the survival was closely linked to lower PCI values and negative cytology, suggesting that tumor biology and baseline disease burden are important determinants for the outcome [58]. This study demonstrates the essential role of complete tumor resection as a key prognostic factor.

The PSOGI multi-institutional cohort [58] similarly reported long-term survivors among 448 patients, with 6.3% living longer than 5 years and occasional survival beyond a decade [59]. The study demonstrated that precise patient selection and expertise are crucial for achieving optimal outcomes. The results confirm that even with aggressive tumor debulking, significant long-term survival can be achieved. These findings support the use of combined surgical and chemotherapeutic approaches in specialized centers.

The GASTRIPEC-I trial represents the only randomized phase III study specifically evaluating the addition of HIPEC to CRS in gastric cancer with synchronous peritoneal metastases [8]. Although the overall survival was comparable in both treatment arms, significant differences in progression-free survival and metastasis-free survival were observed in the group receiving additional HIPEC (7.1 vs. 3.5 months, *p* = 0.047; and 10.2 vs. 9.2 months, *p* = 0.0286, respectively). However, interpretation of these results is limited by several factors, including the prolonged recruitment, premature trial termination, limited statistical power, and heterogeneity in systemic chemotherapy regimens. These findings suggest that, with careful patient selection, the addition of HIPEC may lead to delaying disease progression. At the same time, the study highlights the need for further investigations to better define the long-term benefit of this combined approach (Table 1).

The PERISCOPE II trial offers one of the most critical counterpoints to aggressive surgical approaches. Research data from different studies focused on Asia suggested a better outcome in patients with disseminated gastric cancer after CRS and HIPEC [60]. In this context, a Dutch Research group initiated the prospective PERISCOPE II Trial to investigate the outcome and morbidity of gastrectomy followed by CRS and HIPEC in locally advanced gastric cancer in contrast to chemotherapy alone [10]. Prior to the PERISCOPE II trial, the PERISCOPE I trial showed good feasibility and tolerance of preoperative chemotherapy followed by gastrectomy and CRS/HIPEC in patients with peritoneal metastasis and gastric cancer [61]. This therapy regime was the foundation for the surgical intervention group in Periscope II. In this study, the authors compared, in a randomized controlled trial, ongoing chemotherapy vs. gastrectomy and CRS/HIPEC after initial chemotherapy in locally advanced gastric cancer with PCI < 7. 102 patients were included and randomized to both therapy groups. The study recruitment ended prematurely due to interim analysis. The authors showed comparable survival (17.3 vs. 15.6 months, *p* = 0.12) combined with an elevated SAE rate in the surgery group (44% vs. 8%) with three treatment-related deaths in the surgery group [62]. Because of no survival benefit and elevated morbidity and mortality, gastrectomy followed by CRS/HIPEC is not generally recommended in gastric cancer patients with peritoneal carcinomatosis and should be reserved for special cases and studies.

Treatment of metastatic gastric cancer is associated with substantial morbidity. Systemic therapy is known to result in a high rate of severe adverse events, mainly diarrhea and long-term neuropathy (see Table 1). Likewise, surgical approaches are associated with complications such as postoperative ileus, anastomotic insufficiency, and surgical site infections, which may necessitate reoperation. The mortality rates reported in randomized surgical trials range from 1% to 6% (see Table 2). The results of the retrospective cohort study PRECISE-SG1 demonstrated that a multimodal treatment approach consisting of bidirectional chemotherapy and CRS plus HIPEC offered significant survival benefits in a cohort of 42 patients [63]. The data emphasize that an individually tailored therapy can optimize treatment outcomes. In addition to improved survival, a favorable safety profile was reported.

These findings provide preliminary evidence that an aggressive treatment strategy is feasible in a selected patient population but should be interpreted with caution, as they are currently based on abstract-only reporting.

However, these procedures are associated with considerable perioperative burden, including relevant morbidity and a risk of treatment-related mortality. In PERISCOPE II, higher rates of serious adverse events were observed in the surgical arm [62].

In addition, prolonged recovery may affect the timely delivery of systemic therapy, and the quality of life can be temporarily impaired. As most available data derive from highly selected populations treated in expert centers, the generalizability of these findings to routine clinical practice remains uncertain. Therefore, CRS/HIPEC should be considered an individualized treatment option in selected patients and preferably applied within specialized multidisciplinary programs and prospective clinical trials.

### 8.3. Intraperitoneal Chemotherapy: PIPAC

Pressurized intraperitoneal aerosol chemotherapy (PIPAC) shows potential to improve the survival and quality of life in patients with gastric cancer and peritoneal metastasis, though further large-scale trials are needed. A meta-analysis of 18 studies (671 patients, 1357 procedures) found that 32.6% completed three or more treatments, with a histological response rate of 66.3%. Reduced ascites occurred in 13.1%, and 7.8% of patients became resectable. Adverse events affected 17.1% of patients, with severe events in 3.6%, and PIPAC-related mortality was 0.1%. The overall survival rates for 2 years were 20.0%, with a median survival of 11.7 months [63]. Better outcomes were seen in patients receiving prior or concurrent systemic therapy and multiple PIPAC sessions. PIPAC has the potential to improve the survival and quality of life in patients with gastric cancer and peritoneal metastasis, but further large-scale trials are needed.

### 8.4. Normothermic Intraperitoneal Chemotherapy: NIPS

Interest is shifting toward systemic combined with intraperitoneal therapies. Data from the randomized, controlled, and multicenter DRAGON-01 trial presented at ASCO GI 2025 evaluated an innovative bidirectional chemotherapy approach incorporating both intraperitoneal and intravenous paclitaxel in combination with oral S-1, compared with a control arm receiving palliative intravenous/oral chemotherapy only [9]. The previously reported results, based on an interim analysis of 222 enrolled patients, suggest that this approach may improve the overall survival in patients with peritoneal metastases (19.4 vs. 13.9 months; *p* = 0.005). An acceptable toxicity profile was also observed, indicating good tolerability. This study provides important impulses for future treatment strategies. Conversion surgery was possible if re-laparoscopy revealed an obvious shrinkage of peritoneal metastases, the peritoneal cytology was negative, no other distant metastases were present, the primary was resectable, and the patient maintained a good performance status.

## 9. Conclusions

Peritoneal metastatic gastric cancer remains a predominantly palliative condition, characterized by a poor overall prognosis and significant diagnostic and therapeutic challenges. Systemic therapy continues to represent the standard of care; however, its benefit in patients with isolated peritoneal disease is difficult to quantify, as this subgroup is underrepresented in pivotal trials, and response assessment remains challenging. The combination of chemotherapy and/or immunotherapy with intraperitoneal treatment modalities, such as PIPAC or NIPS, enables direct therapeutic delivery and allows for the macroscopic and histological assessment of the treatment response. Carefully selected patients with a low peritoneal tumor burden, favorable tumor biology, stable disease, and a realistic likelihood of achieving complete cytoreduction may be considered for multimodal treatment in specialized centers. Nevertheless, the current evidence supporting cytoreductive surgery (CRS), hyperthermic intraperitoneal chemotherapy (HIPEC), and other intraperitoneal approaches remain heterogeneous, limited to selected populations, and partly preliminary. Rigorous staging, multidisciplinary evaluation, and well-designed prospective studies are essential to better define which patients may derive meaningful benefit.

## Figures and Tables

**Table 1:** Studies specifically address gastric cancer with peritoneal metastases.

Author	Study Name	Study Design	Molecular Required Pathology	PCC	HER2+	PDL1	MSS	CLDN 18.2	M1 PER	PCI	SAE > 2	Mortality	mOS (mo)	mPFS (mo)
Bang [2]	ToGA	RCT, Phase III	HER2+	na	100%	na	na	na	na	na	32%	3%	13.8	6.7
Al-Batran [3]	AIO FLOT3	prospective multicenter Phase II	na	na	na	na	na	na	7%	na	na	na	na	na
Janjigian [4]	CheckMate 649	randomized multicenter Phase III	PDL1 > 5	18%	0%	16%	88%	na	38%	na	16%	1%	14.4	0.1
Shitara [5]	Spotlight	randomized multicenter Phase III	CLDN18.2+	na	0%	13%	na	100%	na	na	87%	2%	18.2	10.6
Janjigian [6]	KeyNote 811	randomized multicenter Phase III	HER2+	na	100%	85%	93% (2% MSH-H)	na	na	na	71%	1%	28.4	10.0
Al-Batran [7]	RENAISSANCE (AIO-FLOT5)	randomized multicenter Phase III	na	na	na	na	na	na	29%	na	na	na	23.6	na

PCC: poorly cohesive carcinoma; HER2: Human Epidermal Growth Factor Receptor; PDL1 CPS: Programmed Death-Ligand 1 Combined Positive Score; MSS = pMMR: microsatellite stable or proficient mismatch repair; CLDN 18.2: Claudin 18.2; PCI: peritoneal cancer index; SAE: severe adverse events; na: not available.

**Table 2:** Studies specifically address gastric cancer with peritoneal metastases and intraperitoneal chemotherapy.

Author	Study Name	Study Design	Molecular Required Pathology	HER2+	PDL1	MSS	CLDN 18.2	M1 PER	PCI	SAE > 2	Mortality	mOS (mo)	mPFS (mo)
Rau [8]	GASTRIPEC	randomized multicenter Phase III	na	8%	na	na	na	100%	1–39	46%	3%	14.9	7.1
Yan [9]	DRAGON	randomized multicenter Phase III	na	na	na	na	na	100%	1–39	na	0%	19.4	na
Koemans [10]	PERISCOPE II	randomized multicenter Phase III	na	na	na	na	na	100%		48%	6%	15.2	na

PCC: poorly cohesive carcinoma; HER2: Human Epidermal Growth Factor Receptor; PDL1 CPS: Programmed Death-Ligand 1 Combined Positive Score; MSS = pMMR: microsatellite stable or proficient mismatch repair; CLDN 18.2: Claudin 18.2; PCI: peritoneal cancer index; SAE: severe adverse events; na: not available.

## Data Availability

See the cited literature in the article.

## Abbreviations

The following abbreviations are used in this manuscript:

AJCC	American Joint Committee on Cancer
EMA	European Medicines Agency
FAPI	Fibroblast Activation Protein Inhibitor
FDA	Food and Drug Administration
FDG	Fluorodeoxyglucose
PET	Positron Emission Tomography
